# Exploring the impact of public health-related academic competitions on the competencies of university students: evidence from Anhui Province

**DOI:** 10.3389/fpubh.2025.1600566

**Published:** 2025-06-18

**Authors:** Qiancheng Zhou, Shengyan Hu, Chenxi Nian

**Affiliations:** ^1^School of Public Administration, Jilin University, Changchun, China; ^2^School of International Trade and Economics, University of International Business and Economics, Beijing, China; ^3^School of Law, Central China Normal University, Wuhan, China

**Keywords:** public health, academic competitions, competencies of university students, public health education, education innovation

## Abstract

**Background:**

Since the outbreak of COVID-19 in 2019, public health emergencies have garnered widespread attention, leading to an increasing emphasis in academia on cultivating high-quality public health professionals. In the context of digital transformation, public health competitions have emerged as an innovative educational approach for developing multifaceted public health talent.

**Methods:**

This study examines the current state of university students’ participation in public health competitions in Anhui Province, employing a mixed-methods approach that combines qualitative and quantitative analyses, including SWOT and PEST models, Pearson correlation analysis, cross-tabulation analysis, and regression analysis, to assess the impact of public health competitions on students’ abilities.

**Results:**

Firstly, the overall environment for students’ participation in public health discipline competitions is favorable, with more advantages than disadvantages and more opportunities than challenges. Secondly, the main reason why college students are willing to participate in these competitions is the job - hunting advantages they offer, while the primary reason for not participating is a lack of self - confidence. Thirdly, competitions have a significant effect on enhancing college students’ comprehensive abilities, particularly in areas such as psychological coordination ability and the ability to learn medical knowledge and skills. Finally, the frequency of participation, the relevance between teaching and competition, and the enthusiasm for participation have a significant impact on the effectiveness of the competitions.

**Conclusion:**

Based on these findings, this paper offers corresponding recommendations to further improve the effectiveness of public health competitions, aiming to provide theoretical guidance for their promotion and optimization, thereby advancing the innovation and development of public health education.

## 1 Introduction

In the context of frequent global public health events such as COVID-19, training high-quality public health professionals has become an important task for higher education. Public health discipline competitions have gradually emerged in Chinese universities as an innovative educational model in recent years (1). They not only provide students with a platform to showcase their professional skills but also stimulate their learning interest and professional competence through competitive formats that emphasize practicality, innovation, and teamwork (2). Additionally, with the acceleration of globalization and urbanization, public health issues have become increasingly complex, leading to a growing demand for public health professionals. Traditional models of public health education primarily focus on the impartation of theoretical knowledge while inadequately fostering students’ practical abilities, innovative capacities, and teamwork skills (3, 4). The emergence of public health discipline competitions offers a new perspective and approach to addressing this issue.

In recent years, the role of discipline competitions in higher education has garnered increasing attention. Research indicates that such competitions can effectively enhance students’ professional skills and strengthen their innovation and practical abilities (5, 6). In the field of public health, a significant number of students participate in competitions that simulate real-world public health challenges (7). Since the outbreak of COVID-19, universities in Anhui Province have placed a high emphasis on public health education and actively organized related competitions, such as the Anhui Medical University Public Health Competition, Anhui Provincial Health and Wellness System Vocational Skills Competition, Bengbu Medical University Student Public Health Technical Skills Contest, and Anhui Medical Popular Science Contest (8, 9). Additionally, Anhui university students have actively engaged in national-level public health competitions, including the New Era Health Science Popularization Works Collection Contest, China Health Science Popularization Competition, Wuhan Public Health Contest, Student Public Health Science Popularization Challenge, and the National College Student HIV Prevention Knowledge Competition (10, 11). These events encourage students to apply theoretical knowledge to practice, thereby improving their problem-solving skills. Moreover, team-based competitions foster teamwork spirit and communication skills (12, 13). However, systematic research on the impact of public health discipline competitions on students’ skill development remains limited, particularly in China, where certain regions face pronounced poverty and underdevelopment, and educational levels are relatively lagging (14).

Based on our field investigation, participation in public health discipline competitions by university students yields multiple positive outcomes (15, 16). Engaging in these competitions involves a comprehensive and multi-faceted process, not merely passive participation but active engagement in thinking expansion, innovation enhancement, and team communication and collaboration (17, 18). Through this process, students improve their public health skills, increase their knowledge literacy, and develop team awareness. According to our field surveys and interviews, the vast majority of students reported significant gains after participating in these competitions (19–21).

This research investigates the public health discipline competition data from several universities in Anhui Province, assessing the competitions’ effects on students’ emergency response capabilities, teamwork and communication abilities, learning of medical knowledge and skills, psychological coordination capabilities, and innovative thinking abilities through a mixed-methods approach (22, 23). This study not only aids in understanding the mechanism by which discipline competitions contribute to public health education but also serves as a reference for curriculum design and teaching reform in public health majors in higher education (24, 25). Moreover, it aims to explore regional and institutional variations in the effectiveness of public health discipline competitions, providing scientific evidence for the rational allocation and optimization of educational resources (26, 27).

**Figure 1:**
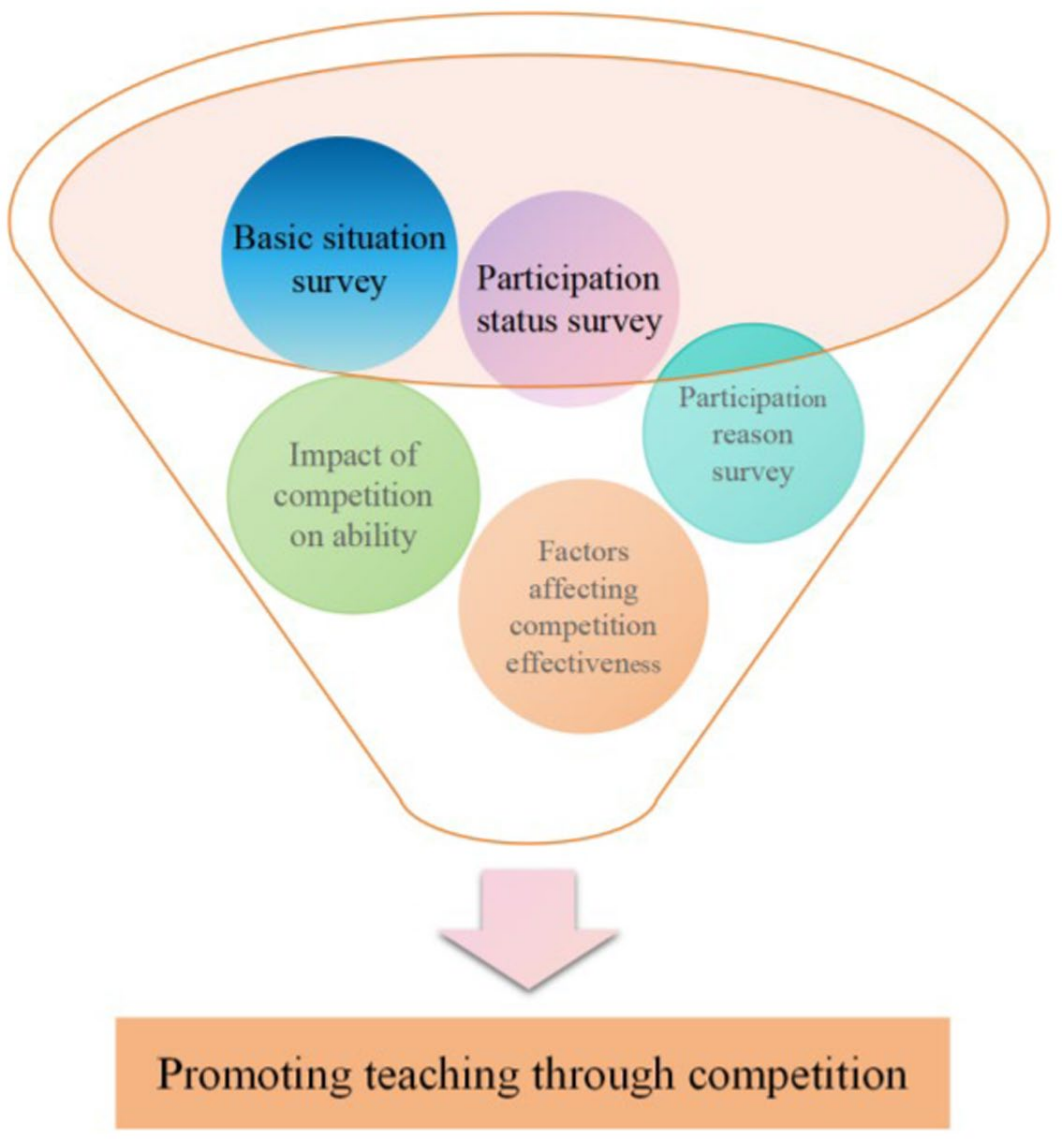
Research framework diagram.

The purpose of this paper is to empirically explore the potential of public health discipline competitions in enhancing university students’ abilities (28). Specific research questions include: the status of university student participation in public health discipline competitions, the factors influencing students’ willingness to participate, the current state of participation (29–31), the impact of public health discipline competitions on students’ abilities, and the factors affecting the effectiveness of competitions for university students (32, 33). According to the above survey and research questions, the research framework of this paper is shown in Figure 1. By conducting field surveys and analyzing these issues, this paper hopes to provide scientific evidence for the promotion and optimization of public health discipline competitions, further advancing innovation and development in public health education. Through investigating the potential of public health discipline competitions to enhance students’ abilities, this research provides new ideas and methods for cultivating high-quality public health professionals (34). Additionally, it aims to offer reference points for curriculum design and teaching methods in public health majors and provide a scientific basis for the rational allocation and optimization of educational resources (35).

## 2 Materials and methods

### 2.1 Study area

**Figure 2:**
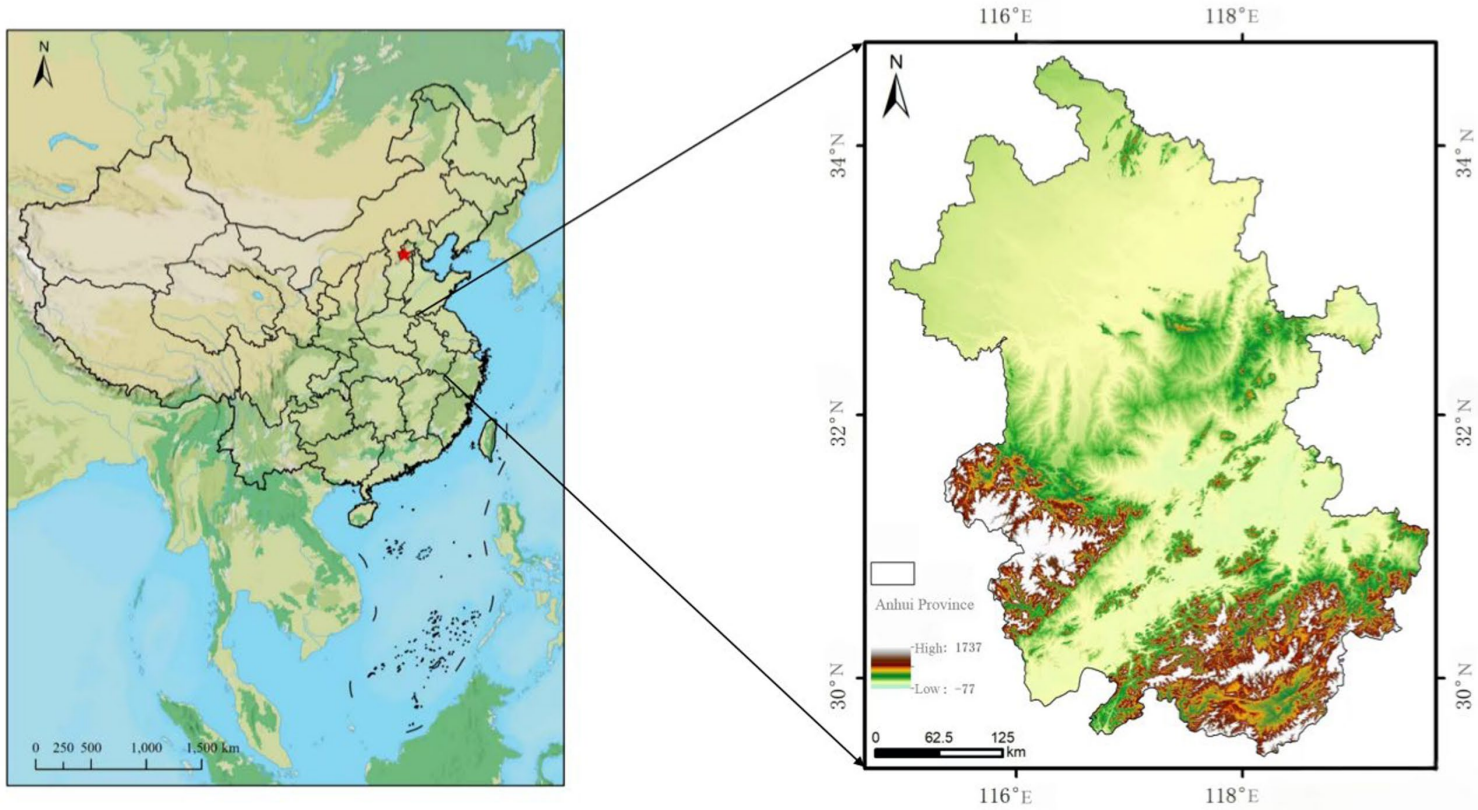
Location map of Anhui Province.

This study uses a survey from Anhui Province as an example. Located in East China, within the Yangtze River Delta region (Figure 2), Anhui Province spans an area of 140,100 square kilometers and includes 16 prefecture-level cities, with a permanent population of approximately 61.23 million. Anhui is one of the important birthplaces of prehistoric civilization in China, boasting a rich cultural heritage. Furthermore, Anhui is a crucial component of the Yangtze River Delta economic region, facilitating maritime access and situated at the strategic intersection for national economic development and several major domestic economic zones. In 2024, Anhui Province’s gross regional product (GRP) reached 5,062.5 billion yuan, reflecting a growth of 5.8% compared to the previous year.

Anhui Province has extensive practical experience and a strong academic foundation in the field of public health. This wealth of practical experience and academic achievements provides rich case studies and resources for public health competitions, making the competition content more relevant to real-world situations and inherently more challenging. Additionally, Anhui Province also has a high level of higher education, with several well-known universities that excel in teaching and research in the field of public health. Lastly, the province has achieved notable successes in public health competitions, with its universities actively participating and frequently gaining commendable results. Thus, selecting Anhui Province as a case study to explore the impact of public health competitions on college students’ abilities is representative and offers deeper insights into the role and value of these competitions in cultivating students’ competencies in public health.

### 2.2 Sample size determination

This survey employed simple random sampling. Based on the sample size calculation formula, with a confidence level of 98% (Z = 2.3263), a margin of error (E) of 3%, and a probability (P) of 0.5, the required sample size (N) is calculated to be 1,503. To ensure that the survey meets expectations and maintains data integrity, the minimum sample size should be at least 1,503. However, to enhance the authenticity and validity of the data collection, as well as to uphold the rigor and scientificity of the research findings, we plan to collect 3,000 questionnaires.


$$N=Z^{2}*[P*(1-P)]/E^{2}$$

### 2.3 Data collection

This data survey focused primarily on public undergraduate universities within Anhui Province. The specific participants were full-time enrolled students with official Chinese accreditation, including undergraduate students, master’s, and doctoral candidates. Data collection was conducted through offline team efforts, supplemented by online surveys utilizing big data mining techniques. The target was to gather 3,000 questionnaires; ultimately, 3,500 questionnaires were distributed, and after removing invalid data, a total of 3,245 valid questionnaires were recovered.

This research primarily involved field surveys and data collection from universities in Anhui Province. The surveyed institutions included Anhui Medical University, Anhui University of Science and Technology, Huainan Normal University, Bengbu Medical University, Anhui University of Chinese Medicine, University of Science and Technology of China, Hefei University of Technology, Anhui University, Anhui Jianzhu University, Anhui Agricultural University, Anhui Normal University, Anhui University of Science and Technology, Huaibei Normal University, and other well-known universities within the province. Given that the data collection combined both online and offline methods, online surveys were primarily disseminated through targeted distribution via students from these institutions, while offline surveys were conducted by our team members through on-site investigations.

### 2.4 Research methods

(1) SWOT and PEST model analysis.

The SWOT analysis, also known as strength-weakness analysis, is a strategic planning tool that evaluates an organization’s strengths, weaknesses, opportunities, and threats in a competitive context. The PEST analysis focuses on macroenvironmental factors, where P stands for Political, E for Economic, S for Social, and T for Technological. The PEST model provides an effective understanding of the broader context, facilitating macro-level decision-making.

(2) Reliability and validity analysis.

Reliability refers to the degree of consistency of results obtained through repeat measurements on the same subject using the same method. Reliability indicators are often expressed as correlation coefficients, with the Cronbach’s alpha being the most commonly used reliability coefficient.

Validity analysis assesses the accuracy of measurement standards of the scale. A KMO value above 0.8 indicates good validity; values between 0.7 and 0.8 suggest acceptable validity; values between 0.6 and 0.7 reflect moderate validity; and values below 0.6 indicate poor validity.

(3) Pearson correlation analysis.

The Pearson correlation coefficient measures whether two data sets align linearly. It assesses the linear relationships between interval variables. When both variables are normally distributed and continuous, their relevant degree of correlation is expressed as the product–moment correlation coefficient, specifically the Pearson simple correlation coefficient.

(4) Cross-analysis.

Cross-analysis, also known as dimensional analysis, builds on both longitudinal and cross-sectional methods to analyze relations from a cross-sectional and three-dimensional perspective, progressing from shallow to in-depth analysis. This method is typically used to explore relationships between two variables, designating one as the row variable and the other as the column variable, allowing for flexibility in the number of variables involved.

(5) Regression analysis.

In statistics, regression analysis refers to a statistical method used to determine the quantitative relationship between two or more variables that depend on each other. This paper employs regression analysis to verify the factors affecting the effectiveness of college students’ participation in academic competitions. Regression analysis can accurately quantify the strength and direction of these variable relationships, directly aligning with research objectives and providing answers to key questions.

## 3 Basic information statistics

### 3.1 Sex information statistics

According to the basic gender information statistics in this survey, men accounted for 47.46%, and women accounted for 52.54%. Overall, there were slightly more women than men.

### 3.2 Grade information statistics

**Figure 3:**
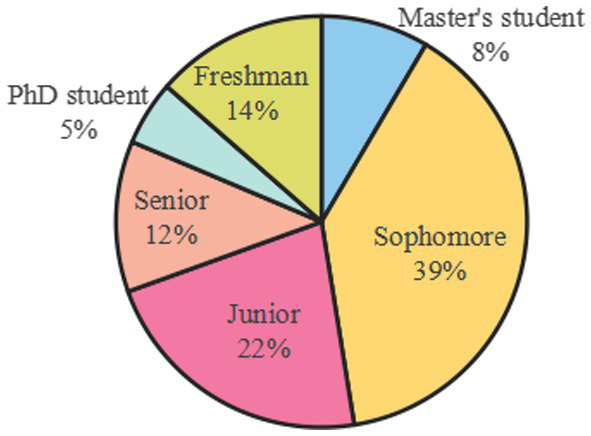
Grade information statistics.

According to the statistical information of grade (Figure 3), the freshmen accounted for 13.56% of the total number, 38.98% of the sophomore year, 22.03% of the junior year, 11.86% of the senior year, master students and doctoral students accounted for 8.48 and 5.09% of the total number, respectively. The sophomore year accounted for the highest proportion of the total number of students, followed by the junior year and the freshman year. According to the data, sophomore students are the most motivated to participate in the public health discipline competition.

### 3.3 Professional information statistics

**Figure 4:**
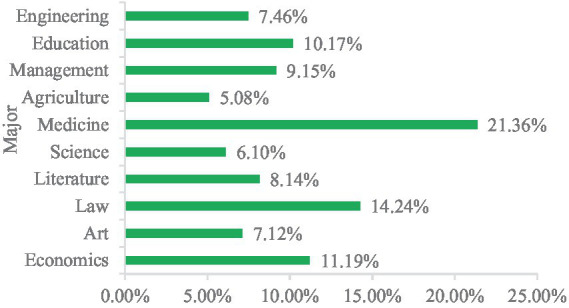
Professional information statistics diagram.

The survey statistics of 10 professional categories of students (Figure 4), including the most professional for medical class 21.36%, followed by law and economy of 14.24 and 11.19%, can reflect the public health competition main students professional status, also help us to understand the enthusiasm of different professional students to participate in public health discipline competition.

## 4 Analysis results

### 4.1 Investigation on the current situation of university students’ participation in public health discipline competitions

**Figure 5:**
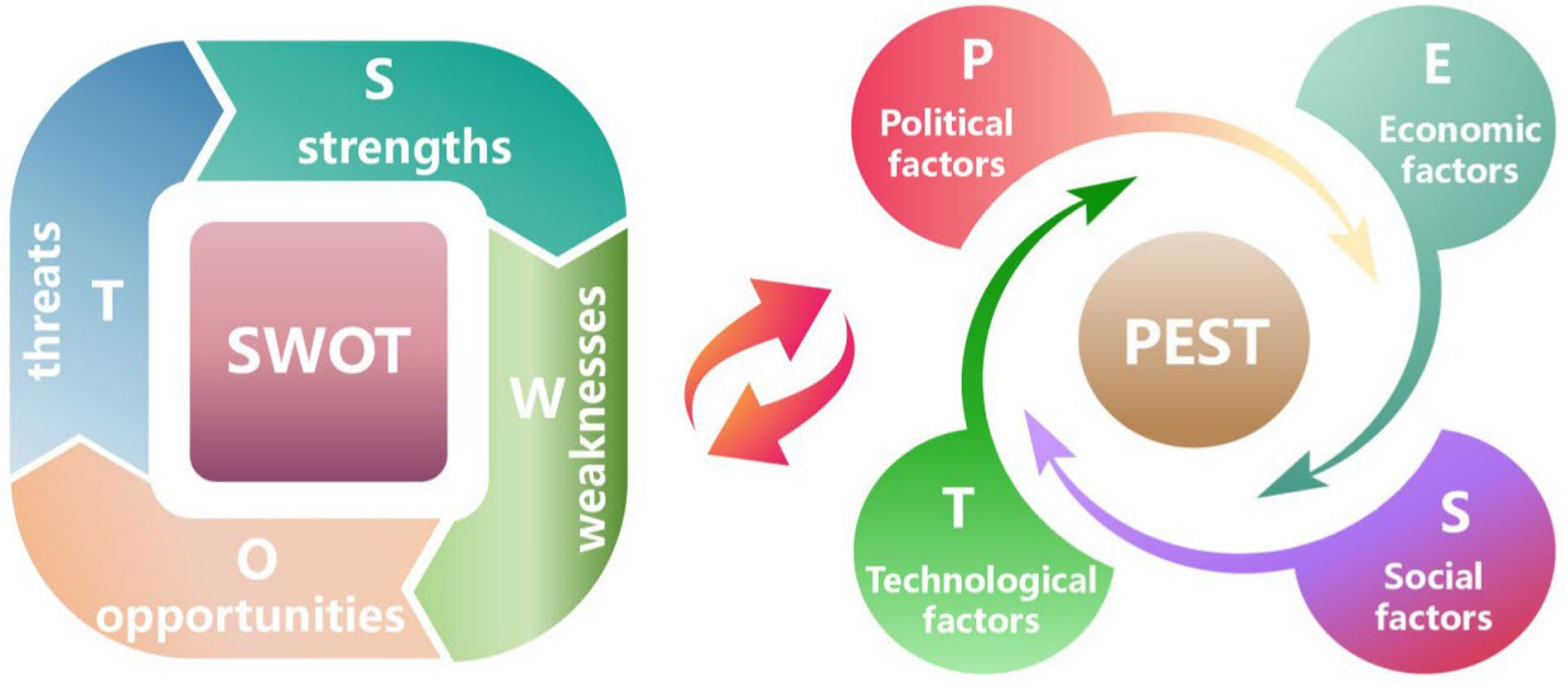
SWOT and PEST model diagram.

In order to further investigate the basic background and environment of college students’ participation in subject competitions, we conducted SWOT and PEST model analysis (Figure 5).

(1) SWOT model analysis.

Through SWOT model, we can effectively help us visualize the advantages and disadvantages, opportunities and challenges of college students’ participation in public health discipline competitions.

Strengths: High level of attention to academic competitions; extensive support from institutions; Internet technology provides technical support for students.

Weaknesses: Long preparation cycles and high energy consumption; some students lack confidence; if they do not win an award, it may lead to feelings of depression.

Opportunities: Society places high importance on talent; universities have increased their support and encouragement; academic competitions have become more open and diversified.

Threats: Traditional teaching models have drawbacks; increased requirements for instructors; Increased requirements for students.

(2) PEST model analysis.

PEST model analysis can help us understand the political environment, economic environment, social environment and technical environment of college students participating in discipline competition.

Political factors: The country emphasizes the cultivation of public; policies have been introduced to encourage students to participate in public health competitions; competitions have been incorporated into practical teaching.

Economic factors: The overall economic environment of the country is stable; the proportion of funding allocations for the public health industry has increased; numerous universities support the development of public health education.

Social factors: Society has formed a positive atmosphere of valuing public health; the integration of teaching and competition has become a social consensus; social capital has collaborated with universities to conduct various public health education activities.

Technological factors: New technologies, such as ChatGPT, Deepseek, and other artificial intelligence tools, have emerged; academic competitions serve as an effective guarantee for talent cultivation; new technologies provide support for students.

(3) Summary of SWOT and PEST analyses.

From the above analysis, it can be concluded that, overall, the advantages of college students participating in disciplinary competitions outweigh the disadvantages, and opportunities surpass challenges. Secondly, the government has increased support for public health disciplinary competitions, promoting their development toward greater openness and diversity. However, there are still several issues to address in the current educational models of these competitions, which pose a challenge to traditional teaching methods. Thirdly, the environment for college students to participate in public health competitions is favorable. From political, economic, social, and technological perspectives, a supportive environment is evident, reflecting society’s strong desire for talented individuals. As university students, they should actively participate in these competitions to discover their potential, improve their skills, and achieve the goal of “promoting education through competitions.”

### 4.2 Survey on university students’ willingness to participate in public health discipline competitions

(1) Reasons for student participation in competitions.

**Figure 6:**
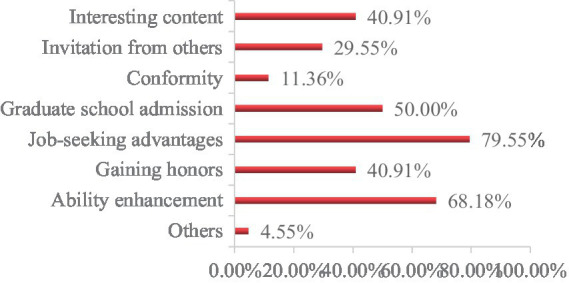
Reasons for university students to participate in the competition.

According to the survey results (Figure 6), the primary reason for participating in competitions is to “gain a competitive advantage in job hunting,” accounting for 79.55%, which is the highest proportion. The second reason is to “enhance personal abilities,” with a proportion of 68.18%, followed by “graduate school admission,” with a proportion of 51.69%.

(2) Reasons why college students do not participate in the competition.

**Figure 7:**
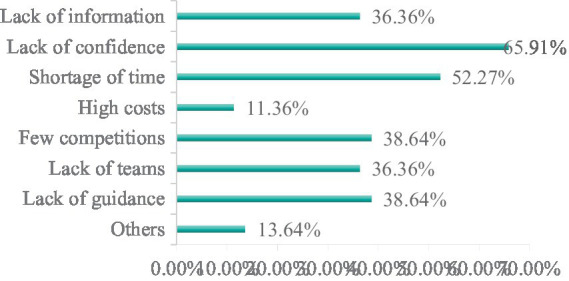
Reasons why college students did not participate in the discipline competition.

According to the survey results (Figure 7), 65.91% of the students did not participate in the competition due to lack of confidence, and 52.27% did not participate in the competition due to lack of time.

### 4.3 Reliability and validity analysis

We conducted a reliability analysis of the obtained questionnaire (Table 1). The analysis finds that the reliability coefficient value is 0.829, greater than 0.8, which indicates that the reliability quality of the research data is very high and meets the expected requirements, and follow-up data analysis can be carried out.

**Table 1:** The reliability analysis table.

**Cronbach** Reliability analysis		
Number of terms	Sample capacity	Cronbach α
55	3,245	0.829

We conducted the validity analysis of the collected data (Table 2). According to the results, the KMO value is 0.876, and the KMO value is greater than 0.8, which means that the validity of the study data is very good.

**Table 2:** Validity analysis table.

KMO and Bartlett		
Sample a sufficient Kaiser-Meyer-Olkin metric.		0.876
The sphericity test of the Bartlett	Approximate chi square	32460.012
	df	1,276
	Sig.	0.000

### 4.4 Exploration of the effectiveness and role of public health discipline competitions

By investigating university students’ overall recognition of their participation in academic competitions, we can clearly and transparently demonstrate their acknowledgment of public health discipline competitions. Data reveals that a total of 68.29% of respondents believe that discipline competitions provide substantial or considerable assistance to university students, while 29.27% perceive the impact as moderate. Only 2.44% of students report that these competitions provide minimal or very little help.

Building on relevant literature and the measurement scale of competition’s impact on student abilities (36, 37), this study employs Pearson correlation analysis to examine the effect of public health competitions on five student competencies: emergency response capability, teamwork and communication ability, medical knowledge and skill acquisition, psychological coordination ability, and innovative thinking ability. The results are presented in Table 3.

**Table 3:** Pearson correlation analysis.

Competencies of university students	Public health discipline competition	
Emergency response capacity	Pearson Correlation	0.886**
	Significance (Bilateral)	0.000
Team and communication skills	Pearson Correlation	0.887**
	Significance (Bilateral)	0.000
Medical knowledge and skills learning ability	Pearson Correlation	0.887**
	Significance (Bilateral)	0.000
Psychological coordination ability	Pearson Correlation	0.897**
	Significance (Bilateral)	0.000
Innovative thinking ability	Pearson Correlation	0.882**
	Significance (Bilateral)	0.000
Sample capacity N		3,245

**Indicates *p* < 0.01, that the correlation is more significant at the 99% confidence level.

Based on the Pearson correlation coefficients and significance values, it is evident that there is a significant positive correlation between student participation in public health discipline competitions and the enhancement of their abilities. Furthermore, the correlation with psychological coordination skills, teamwork and communication abilities, medical knowledge and skill acquisition, emergency response capability, and innovative thinking ability decreases in that order.

To ensure the authenticity and effectiveness of the survey results, our team conducted interviews with students and teachers who participated in public health discipline competitions based on the questionnaire data (Table 4). The interviews revealed a significant consistency between the descriptions provided by teachers and students and the results of the field survey, indicating that public health discipline competitions play a crucial and positive role in enhancing relevant student capabilities.

**Table 4:** Interview survey table.

Respondent	Individual status	Interview questions	Method
Mengru Dai	Master’s student of Southeast University	The role of public health competition on their own innovative thinking ability.	Field interview
Zixuan Wang	Undergraduate student of Anhui Normal University	Impact of public health competitions on the ability to learn medical knowledge and skills.	Field interview
Xianghui Liu	Master student of Anhui University	The role of public health competitions on mental coordination ability.	Field interview
Xiangfei Guo	Undergraduate student of Anhui University of Science and Technology	The role of public health contests on emergency response capacity.	Online interview
Weijia Ge	Teacher of Xinyang University	As the instructor, have students improved the above five abilities in public health competitions by leading them to participate in public health competitions?	Field interview
Xiaoning Zhang	Master’s student from Monash University	Public health competition for teamwork and communication skills.	Online interview

### 4.5 Analysis of factors affecting the effectiveness of public health competitions

To further investigate the reasons for the influence of students’ achievements in the public health discipline competition, this study investigated the three key factors of the competition based on the reference of relevant literature (38, 39), this study examines three key factors: frequency of participation, relevance of the competition to teaching, and student enthusiasm for participation.

Using the variables “frequency of participation,” “relevance of teaching to competition,” and “enthusiasm for participation” as grouping items, taking the degree of assistance provided by public health discipline competitions as the dependent variable, a cross-tabulation analysis was conducted (Table 5). The results, based on the Chi-square values and significance *p*-values of 0.000***, 0.000***, and 0.017**, demonstrate significant levels, leading to the rejection of the null hypothesis. Therefore, there are correlations between the three factors—frequency of participation, relevance of the competition to teaching, and enthusiasm for participation—and the degree of assistance provided by public health discipline competitions.

**Table 5:** Cross-tabulation analysis.

Independent variable	Competition effect	
	χ^2^	*p*
Frequency of participation	95	0.000***
Relevance of teaching to competition	103	0.000***
Enthusiasm for participation	12	0.017**

In order to ensure the accuracy of causality, we further verified the relationship effects between “frequency of participation,” “relevance of teaching to competition,” “enthusiasm for participation” and “the degree of assistance provided by public health discipline competitions.” We further verified the above results through regression analysis (Table 6).

**Table 6:** Regression analysis results.

**Regression analysis results**									
Influencing factors	Non-standardized coefficients		Standardization factor	t	*p*	VIF	R^2^	Adjusted R^2^	F
	B	Standard error	Beta						
Frequency of participation	0.958	0.076	0.857	12.557	0.000***	1	0.734	0.73	*F* = 157.679, *p* = 0.000***
Constant	0.42	0.158	0	2.661	0.010**	–			
Relevance of teaching to competition	0.783	0.047	0.91	16.563	0.000***	1	0.828	0.825	*F* = 274.333, *p* = 0.000***
Constant	−0.204	0.137	0	−1.484	0.143	–			
Enthusiasm for participation	1.011	0.042	0.954	24.071	0.000***	1	0.91	0.909	*F* = 579.411, *p* = 0.000***
Constant	0.146	0.094	0	1.566	0.123	–			

Dependent variable: Do you think the public health discipline competition is helpful to yourself? ***, ** and * represent the significance levels of 1, 5, and 10%, respectively.

According to the regression analysis results, the F-test result shows a significant *p*-value of 0.000***, indicating that the level is significant. Regarding the multicollinearity of variables, all VIF values are less than 10, so there is no multicollinearity issue in the model, and the model construction is sound. “Frequency of Participation,” “Relevance of teaching to competition,” and “Enthusiasm for Participation” are positively correlated with “The Degree of Assistance Provided by Public Health Discipline Competitions.” This means that the higher the frequency of participation, the better the competition outcomes; the stronger the association, the better the competition outcomes; students with high enthusiasm for participation achieve better competition results compared to those with low enthusiasm.

## 5 Discussion

Based on the analysis above, the following points are discussed.

(1) Among the student participants in public health discipline competitions, there are slightly more female participants than male. Additionally, second-year students show the highest enthusiasm for participation in competitions, followed by third-year and first-year students, with doctoral and master’s students exhibiting the lowest levels of participation enthusiasm. Thirdly, among the participating majors, medical majors are the most students, followed by law and economics majors, and the least participating majors are agricultural students.
(2) Based on SWOT and PEST analyses, the overall environment for university students participating in public health discipline competitions is favorable. Firstly, while participation has both advantages and disadvantages, the benefits—such as skill enhancement, broadened horizons, and improved personal competence—generally outweigh the drawbacks, which include long preparation periods and occasional lack of self-confidence. Secondly, student engagement in public health competitions presents opportunities, including societal emphasis on talent development and strong institutional support; however, challenges also exist, such as the limitations of traditional teaching methods and increased demands on educators. Overall, the opportunities far exceed the challenges. Thirdly, political, economic, social, and technological environments strongly support student involvement in these competitions. Increasing funding and resources are being allocated to public health contests, with national-level efforts intensifying support. Universities and various societal sectors actively foster a supportive culture for such competitions. Moreover, advances in big data, artificial intelligence, and other cutting-edge technologies have elevated the standards and quality of public health competitions.
(3) The main motivations for university students to participate in public health discipline competitions include job advantages, skill enhancement, and academic progression. Conversely, the reasons for non-participation include a lack of confidence, time constraints, limited availability of public health competitions, and insufficient guidance.
(4) Public health discipline competitions have a significant positive impact on improving student abilities. Notably, the competitions are particularly effective in enhancing students’ psychological coordination capabilities, followed by medical knowledge and skill acquisition, as well as teamwork and communication abilities. Furthermore, through surveys and interviews, the research team has further substantiated the role of public health discipline competitions in enhancing student competencies.
(5) The frequency of participation, the relevance of teaching to competitions, and student enthusiasm can significantly influence the effectiveness of public health discipline competitions. The frequency of participation shows a significant positive correlation with competition outcomes; the higher the frequency of participation, the better the competition results. Similarly, the relevance of teaching to competitions is significantly positively correlated with competition effectiveness; the stronger the relevance, the better the outcomes. Students with high enthusiasm for participation achieve superior results compared to those with lower enthusiasm.
(6) Currently, public health discipline competitions are experiencing vigorous growth; however, limited research has focused on their specific impact on university students’ capabilities. Although many universities in Anhui Province organize such competitions extensively, there is a lack of systematic evaluation. This study fills that gap by conducting empirical analysis to explore the mechanisms through which these competitions influence various student skills, including application of professional knowledge, practical operations, and teamwork. The findings not only provide a basis for optimizing competition organization and talent development programs at universities in Anhui but also offer valuable references for institutions across other regions of China. Ultimately, this research aims to contribute to the cultivation of high-quality professionals who meet the evolving demands of public health development and to promote the advancement of public health education.

## 6 Conclusion

According to the survey, the basic characteristics of the respondents align closely with the research findings. Throughout the survey process, the university student population demonstrated a strong interest in participating in public health discipline competitions. Based on this research, the following conclusions and recommendations have been formulated.

### 6.1 Promote broader participation among university students

Encourage students from different grades and majors to engage in public health discipline competitions by establishing multi-tiered and diverse types of competition projects that cater to varying student needs and interests (40, 41). At the same time, enhance the promotion and publicity of these competitions to increase university students’ awareness and participation (42).

### 6.2 Strengthen support from multiple entities

The government should increase funding and policy support for public health discipline competitions, providing necessary guarantees for schools and social organizations (43). Schools should establish a comprehensive competition management system, offering essential faculty and venue support while encouraging students to actively participate in competitions (44, 45). Social organizations should also be actively involved in organizing and sponsoring competitions to provide robust support for their successful implementation (46).

### 6.3 Enhance interdisciplinary competition interaction

Facilitate interaction between public health discipline competitions and competitions in other fields, such as medicine, environmental science, and computer science, by collaborating on interdisciplinary competition events (47, 48). This approach can broaden students’ perspectives and knowledge bases while promoting the cross-disciplinary integration and innovative development of various fields.

### 6.4 Establish a comprehensive feedback mechanism for competitions

Implement a well-structured feedback mechanism to collect opinions from participating students, guiding teachers, and judges to comprehensively assess the planning, organization, implementation, and outcomes of the competitions (49). Based on the feedback, timely adjustments and improvements to competition plans should be made to enhance the quality and standards of the events (50).

### 6.5 Strengthen the transformation and application of competition results

Encourage and support the transformation and application of competition outcomes, promoting outstanding competition entries and research findings into the market or practical application fields (51, 52). By collaborating with businesses, research institutions, and other entities, efforts should be made to drive the industrialization, commercialization, and socialization of competition results, thereby contributing significantly to the development of public health initiatives.

## Data Availability

The datasets presented in this article are not readily available because this is a part of the author’s follow-up research. Requests to access the datasets should be directed to shengyanhu1996@163.com.
